# Neighborhood Predictors of Poor Prenatal Care and Well-Child Visit Attendance

**DOI:** 10.1007/s10995-023-03844-9

**Published:** 2023-11-22

**Authors:** Elizabeth R. Wolf, Alicia Richards, Roy T. Sabo, Steven H. Woolf, Bergen B. Nelson, Alex H. Krist

**Affiliations:** 1 Children’s Hospital of Richmond at VCU, 1000 East Broad Street, Richmond, VA 23219, USA; 2 Department of Pediatrics, Virginia Commonwealth University, Richmond, VA, USA; 3 Department of Biostatistics, Virginia Commonwealth University, Richmond, VA, USA; 4 Department of Family Medicine and Population Health, Virginia Commonwealth University, Richmond, VA, USA; 5 Center on Society and Health, Richmond, VA, USA

**Keywords:** Prenatal care, Child health, Child poverty, Social determinants of health and neighborhood characteristics

## Abstract

**Purpose:**

Women and children continue to miss preventive visits. Which neighborhood factors predict inadequate prenatal care (PNC) and well-child visit (WCV) attendance remain unclear.

**Description:**

In a retrospective case–control study at Virginia Commonwealth University Health System, mothers with less than 50% adherence or initiation after 5 months gestation were eligible as cases and those with ≥ 80% adherence and initiation before 5 months were eligible as controls. Children in the lowest quintile of adherence were eligible as cases and those with ≥ 80% of adherence were eligible as controls. Cases and controls were randomly selected at a 1:2 ratio and matched on birth month. Covariates were derived from the 2018 American Community Survey. A hotspot was defined as a zip code tabulation area (ZCTA) with a proportion of controls less than 0.66. ZCTAs with fewer than 5 individuals were excluded. Weighted quantile regression was used to determine which covariates were most associated with inadequate attendance.

**Assessment:**

We identified 38 and 35 ZCTAs for the PNC and WCV analyses, respectively. Five of 11 hotspots for WCV were also hotspots for PNC. Education and income predicted 51% and 34% of the variation in missed PNCs, respectively; language, education and transportation difficulties explained 33%, 29%, and 17% of the variation in missed WCVs, respectively. Higher proportions of Black residents lived in hotspots of inadequate PCV and WCV attendance.

**Conclusion:**

Neighborhood-level factors performed well in predicting inadequate PCV and WCV attendance. The disproportionate impact impact of inadequate PCV and WCV in neighborhoods where higher proportions of Black people lived highlights the potential influence of systemic racism and segregation on healthcare utilization.

## Introduction

Prenatal care (PNC) attendance is associated with decreased prematurity, (Vintzileos et al., 2002) low birth weight (Gortmaker, 1979) and neonatal mortality (Herbst et al., 2003). Well-child visit (WCV) attendance is associated with reduced hospitalizations and emergency department utilization (Pittard, 2011; Tom et al., 2010). Despite these benefits, many pregnant women and children continue to miss preventive visits. In the United States, 5–12% of pregnant women begin PNC in the third trimester or have no PNC at all (“Late or No Prenatal Care,” 2019). Children 0–5 years miss between a third and half of recommended WCVs (Selden, 2006; Wolf et al., 2018). Studies have shown that poverty, lack of English fluency, lower education, and no insurance are associated with inadequate PNC and WCV attendance, and that inadequate PNC and WCV attendance is more common among people who identify as Black (Alexander et al., 2002; Braveman et al., 2000; Chung et al., 2004; Cox et al., 2011; D'Ascoli et al., 1997; Freed et al., 1999; Frisbie et al., 2001; Jhanjee et al., 2004; Melnikow et al., 1991; Minkovitz et al., 2005; Mustin et al., 1994; Selden, 2006; Wolf et al., 2018). These individual factors tend to cluster together geographically (Kolak et al., 2020).

Richmond, Virginia, where Virginia Commonwealth University Health System (VCUHS) is located, experiences wide geographic health disparities, including those involving maternal and infant mortality (E. Zimmerman et al., 2016a, 2016b). Our goals were to determine if there are also geographic clusters of healthcare underutilization for women and children, and to understand what factors are associated with underutilization.

## Methods

### Settings and Participants

We conducted a retrospective case control study of mothers delivering between May 2017 and May 2018 at VCUHS, an academic safety-net health system. PNC attendance was collected via manual chart review of the electronic health record (EHR), including scanned-in records from outside facilities such as county health departments. WCV attendance was collected electronically for children born at VCUHS 1–3 years of age at the time of data extraction (April 2019) with at least one WCV between 2 and 6 months of age.

### Measurements

We used the Adequacy of Prenatal Care Utilization Index (APCU) to assess prenatal care attendance. The APCU uses the gestational age, the month of initiation and the total number of visits in relation to ACOG recommendations (Guidelines for Perinatal Care, 7th Edition, 2012) to determine whether PNC is adequate (Kotelchuck, 1994). Subjects with inadequate attendance (< 50% observed to expected adherence or initiation ≥ 5 months) were eligible as cases and those with adequate attendance (≥ 80% observed to expected adherence and initiation < 5 months) were eligible as controls. Mothers delivering at < 23-weeks gestation, who were incarcerated, or who had multiple fetuses were excluded because their PNC was expected to fall outside routine obstetric recommendations. WCV adherence was compared to American Academy of Pediatrics (AAP) recommendations for each specific age range (”2015 Recommendations for Preventive Pediatric Health Care Committee on Practice and Ambulatory Medicine and Bright Futures Periodicity Schedule Workgroup,” 2015). We assumed that children did not receive care elsewhere after their last recorded WCV (Wolf et al., 2018). Children with the lowest quintile of adherence were eligible as cases and those with 80% or more adherence were eligible as controls. Children who were born < 35-weeks gestation or who stayed longer than 48 h in the Neonatal Intensive Care Unit were excluded, as they may require more WCVs than AAP standards.

Cases and controls were randomly selected at a 1:2 ratio from eligible subjects and frequency matched on birth month. Patients’ current addresses were geocoded to corresponding zip code tabulation areas (ZCTAs). ZCTAs with < 5 individuals were excluded to avoid the extreme variation associated with low sample sizes. Since there were 2 controls for every case (a proportion of controls of 0.66), we defined a hotspot to be a ZCTA with a proportion of controls < 0.66. ZCTA-level measurements, including median income, education, employment, insurance status, language, race, and transportation were extracted from the 2018 American Community Survey (”American Community Survey,” 2019).

### Statistical Analysis

Bivariate, unadjusted associations between area-level measures and the proportion of controls were estimated through binomial logistic regression (using SAS version 9.4, Cary, NC, USA). Because of the collinearity between the covariates at the neighborhood level, we fit a multivariable model using weighted quantile sum regression (WQS) (Carrico et al., 2015) (using the “wqs” package in R, version 4.0.2) to determine which covariates were most associated with inadequate PNC and WCV attendance. The research was conducted in accord with prevailing ethical principles and reviewed by the VCU Institutional Review Board (Table 1).

## Results

**Fig. 1:**
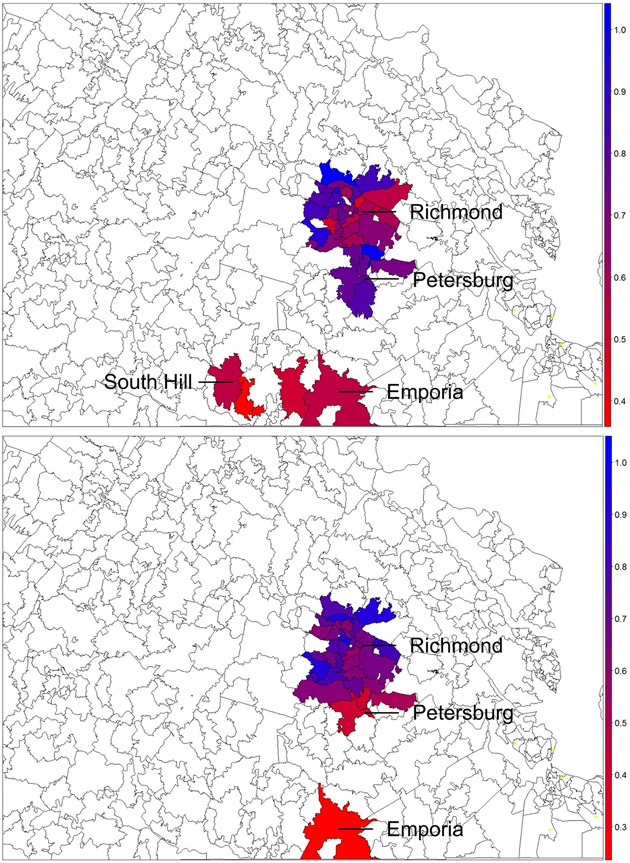
Hotspots of inadequate prenatal care and well-child visits in Central Virginia, with red representing areas of most inadequate attendance and blue representing areas of greatest attendance

We identified 38/86 and 35/74 ZCTAs with 5 or more individuals for the PCV and WCV analyses, respectively; 32 ZCTAs met inclusion criteria for both analyses. Eleven of thirty-eight (29%) ZCTAs were considered hotspots for women with inadequate PNC attendance and 10/35 (29%) were considered hotspots for children with inadequate WCV attendance. Five of 32 (16%) ZCTAs were considered hotpots for both WCV and PNC. The lowest PNC attendance was located in East Highlands (Richmond City), North Chesterfield (Chesterfield County in South Richmond) and South Hill (Mecklenburg County), whereas the lowest WCV attendance occurred in Colonial Heights (Colonial Heights City in North Petersburg), Petersburg (Petersburg City) and Emporia (Emporia City) (Fig. 1).

Neighborhood variables (proportion with lower levels of education, employment, income, transportation, and lack of insurance)—except limited English language—were collinear with Black race. Lower education (51%) and income (34%) explained most of the association with inadequate PNC attendance. Limited English language (33%), lower education (29%) and lack of transportation (16%) explained most of the association with inadequate WCV attendance. With every $10,000 increase in median income, the odds of adequate PNC and WCV attendance increased significantly (1.2 95% CI 1.1, 1.3 and 1.1 95% CI 1.1, 1.2, respectively). A greater proportion of the population with bachelor’s degrees was positively associated with higher odds of adequate attendance for PNC and WCV (77.5 95% CI 11.7, 511.6 and 40.3 95% CI 6.5, 249.4, respectively). A greater proportion of Black residents lived in neighborhoods which had inadequate PCV and WCV attendance.

## Discussion

To our knowledge, this is the first study to examine neighborhood factors associated with both PCV and WCV attendance. About one-half of the identified hotspots were areas of both inadequate PNC and WCV attendance. The concordance between PCV and WCV attendance is consistent with individual-level studies showing that a mother with adequate PNC is almost twice as likely to have a child with adequate WCV attendance (Freed et al., 1999). Several of the hotspots are known to have high infant mortality rates and a large proportion of infants of low birth weight ( Total Infant Deaths by Place of Occurrence and Place of Residence by Race with Resident Infant Death Rates per 1,000 Total Live Births by Planning District and City or County, 2019; Emily Zimmerman et al., 2016a, 2016b). Inadequacy of PNC may help explain these geographic disparities since PNC is associated with greater gestational age and birth weight through control of maternal hypertension, treatment of infectious diseases and identification of anatomic abnormalities (Gortmaker, 1979; Vintzileos et al., 2002).

We found that ZCTAs with lower income, less education, greater unemployment, lack of insurance, lack of English language fluency, and lack of transportation were associated with inadequate attendance of PNC and WCV visits, and that a larger proportion of Black residents lived in ZCTAs with inadequate attendance of PNC and WCV. The collinearity of race with all other predictors of inadequate care (except limited English language) suggests that Black people were more likely to live in neighborhoods that are disproportionally disadvantaged. As in many other cities, the Richmond Metropolitan area has a history of redlining and other structurally racist policies, past and present, that have systematically disadvantaged and segregated people of color and introduced barriers to health care (Rothstein, 2017).

This study represents a first step in reducing disparities in the delivery of high-value care. Health systems do not always have detailed individual level data about their patients but do typically have their address. Conducting geospatial analyses can help health systems identify patients with barriers to care and build facilities or design other interventions to facilitate attendance. Future studies could also employ a Bright Spot approach (Bradley et al., 2009) to identify factors associated with better-than-expected attendance.

The study was subject to several limitations. First, associations at the ZCTA level may not hold true at the individual level. However, our previous study, which examined individual-level data, showed similar findings (Wolf et al., 2020). Second, this study was limited using records of one hospital system and our results may not be generalizable to other settings.

In conclusion, there was a high degree of overlap between several different social determinants of preventive health for pregnant women and children within ZCTAs, much of it associated with race. There may be opportunities to improve preventive visit attendance across two generations by addressing geographically concentrated social determinants of health.

## Figures and Tables

**Table 1:** Odds ratios for prenatal care and well-child visit attendance within zip code tabulation areas in unadjusted and weighted quantile sum regression models

Covariates^a^	Prenatal care (n = 38 zip code tabulation areas representing 891 mothers)				Well child visits (n = 35 zip code tabulation areas representing 833 children)			
	Unadjusted model			Weighted quantile sum regression model	Unadjusted model			Weighted quantile sum regression model
	Odds ratio	95% CI	P value	% of association	Odds Ratio	95% confidence interval	P value	% of association
Median household income per $10,000/year	1.18	(1.10,1.27)	< .001	34.1	1.14	(1.07, 1.22)	< .001	0.7
Education								
High school or less	0.06	(0.02, 0.19)	< .001	51.3	0.08	(0.03, 0.27)	< .001	28.7
Some college	0.004	(< .001, 0.35)	0.02		0.01	(< .001, 0.75)	0.04	
Bachelor’s degree	77.46	(11.73, 511.62)	< .001		40.32	(6.52, 249.37)	< .001	
Graduate degree	328.51	(28.85, > 999.99)	< .001		140.96	(12.41, > 999.99)	< .001	
Employment								
Unemployed	< .001	(< .001, 0.09)	0.004	0.4	< .001	(< .001, 0.01)	< 0.001	11.7
Insured								
Uninsured	0.01	(< .001, 0.08)	< .001	4.2	0.01	(< .001, 0.11)	< 0.001	0.5
Language								
Limited English	0.02	(< .001, 8.26)	0.19	0.2	< .001	(< .001, 0.07)	0.006	33.4
Race								
White	4.28	(2.01, 9.10)	< .001	3.6	2.78	(1.32, 5.82)	0.007	8.6
Black	0.24	(0.12, 0.48)	< .001		0.43	(0.22, 0.84)	0.014	
Other	156.87	(3.92, > 999.99)	0.007		0.68	(0.03, 17.64)	0.82	
Transportation								
No vehicle	0.04	(0.004, 0.36)	0.004	4.1	0.10	(0.01, 0.95)	0.045	16.5

a Covariates derived from the 2018 American Community Survey at the level of the Zip Code Tabulation Area

## Data Availability

Anonymized data is available upon request.

## Significance

### What is already known on this subject?

Many pregnant women and children continue to miss preventive visits. It is not known what geographic factors contribute to missed visits.

### What this study adds?

Neighborhood-level factors performed well in predicting inadequate prenatal care and well-child attendance. There were disproportionate missed preventive visits in neighborhoods where higher proportions of Black people lived, highlighting the role of systemic racism and segregation on healthcare utilization.

## Code Availability

Coding is available upon request.
